# Enhancing [^177^Lu]Lu-DOTA-TATE therapeutic efficacy in vitro by combining it with metronomic chemotherapeutics

**DOI:** 10.1186/s13550-024-01135-0

**Published:** 2024-08-13

**Authors:** Jordan Cheng, Joke Zink, Edward O’Neill, Bart Cornelissen, Julie Nonnekens, Lefteris Livieratos, Samantha Y. A. Terry

**Affiliations:** 1 Department of Imaging Chemistry and Biology, School of Biomedical Engineering and Imaging Sciences, King’s College London, London, SE1 7EH UK; 2 Department of Molecular Genetics, Erasmus MC Cancer Institute, Erasmus University Medical Center, Rotterdam, The Netherlands; 3 Department of Radiology and Nuclear Medicine, Erasmus MC Cancer Institute, Erasmus University Medical Center, Rotterdam, The Netherlands; 4 Nuffield Department of Surgical Sciences, University of Oxford, Oxford, UK; 5 Department of Nuclear Medicine, University Medical Center Groningen, University of Groningen, Groningen, The Netherlands; 6 Department of Biomedical Engineering, School of Biomedical Engineering and Imaging Sciences, King’s College London, London, SE1 7EH UK; 7 Department of Nuclear Medicine, Guy’s & St Thomas’ Hospitals NHS Foundation Trust, London, SE1 7EH UK; 8 Department of Oncology, University of Oxford, Oxford, UK

**Keywords:** Targeted radionuclide therapy, Cell cycle, Cell size, Viability, Combination treatment

## Abstract

**Background:**

Peptide receptor radionuclide therapy (PRRT) uses [^177^Lu]Lu-[DOTA^0^-Tyr^3^]octreotate ([^177^Lu]Lu-DOTA-TATE) to treat patients with neuroendocrine tumours (NETs) overexpressing the somatostatin receptor 2A (SSTR2A). It has shown significant short-term improvements in survival and symptom alleviation, but there remains room for improvement. Here, we investigated whether combining [^177^Lu]Lu-DOTA-TATE with chemotherapeutics enhanced the in vitro therapeutic efficacy of [^177^Lu]Lu-DOTA-TATE.

**Results:**

Transfected human osteosarcoma (U2OS + SSTR2A, high SSTR2A expression) and pancreatic NET (BON1 + STTR2A, medium SSTR2A expression) cells were subjected to hydroxyurea, gemcitabine or triapine for 24 h at 37^o^C and 5% CO_2_. Cells were then recovered for 4 h prior to a 24-hour incubation with 0.7–1.03 MBq [^177^Lu]Lu-DOTA-TATE (25 nM) for uptake and metabolic viability studies. Incubation of U2OS + SSTR2A cells with hydroxyurea, gemcitabine, and triapine enhanced uptake of [^177^Lu]Lu-DOTA-TATE from 0.2 ± 0.1 in untreated cells to 0.4 ± 0.1, 1.1 ± 0.2, and 0.9 ± 0.2 Bq/cell in U2OS + SSTR2A cells, respectively. Cell viability post treatment with [^177^Lu]Lu-DOTA-TATE in cells pre-treated with chemotherapeutics was decreased compared to cells treated with [^177^Lu]Lu-DOTA-TATE monotherapy. For example, the viability of U2OS + SSTR2A cells incubated with [^177^Lu]Lu-DOTA-TATE decreased from 59.5 ± 22.3% to 18.8 ± 5.2% when pre-treated with hydroxyurea. Control conditions showed no reduced metabolic viability. Cells were also harvested to assess cell cycle progression, SSTR2A expression, and cell size by flow cytometry. Chemotherapeutics increased SSTR2A expression and cell size in U2OS + SSTR2A and BON1 + STTR2A cells. The S-phase sub-population of asynchronous U2OS + SSTR2A cell cultures was increased from 45.5 ± 3.3% to 84.8 ± 2.5%, 85.9 ± 1.9%, and 86.6 ± 2.2% when treated with hydroxyurea, gemcitabine, and triapine, respectively.

**Conclusions:**

Hydroxyurea, gemcitabine and triapine all increased cell size, SSTR2A expression, and [^177^Lu]Lu-DOTA-TATE uptake, whilst reducing cell metabolic viability in U2OS + SSTR2A cells when compared to [^177^Lu]Lu-DOTA-TATE monotherapy. Further investigations could transform patient care and positively increase outcomes for patients treated with [^177^Lu]Lu-DOTA-TATE.

**Supplementary Information:**

The online version contains supplementary material available at 10.1186/s13550-024-01135-0.

## Introduction

Neuroendocrine tumours (NETs) are relatively rare and hard to treat. They originate in specialized neuroendocrine cells and can occur anywhere in the body. As 80–100% of gastric NETs overexpress SSTR2A [1], anticancer therapies targeting the SSTR2A receptor remain an excellent strategy. Amongst these, [^177^Lu]Lu-[DOTA^0^-Tyr^3^]octreotate ([^177^Lu]Lu-DOTA-TATE), which binds and irradiates NETs with SSTR2A overexpression, is now a well-established therapeutic option for patients with advanced gastroenteropancreatic NETs. In the landmark Phase 3 clinical trial, NETTER-1, median overall survival was enhanced in NET patients receiving [^177^Lu]Lu-DOTA-TATE from 36 to 48 months, however objective tumour responses were observed in only 18% of patients [2–5]. Therefore, long-term outlook for most patients remains poor and there is still room for improvement. Work is ongoing to enhance the tumour uptake or therapeutic efficacy of [^177^Lu]Lu-DOTA-TATE through, for example, photothermal therapy or combinations with DNA damage repair protein inhibitors or other radiosensitisers [6–11].

As the therapeutic efficacy of peptide receptor radionuclide therapy (PRRT) can still be enhanced, here we investigated an approach combining [^177^Lu]Lu-DOTA-TATE with commonly used chemotherapeutics in NET patients. As well as somatostatin analogues and targeted therapies such as everolimus, and sunitinib, patients with NETs can also receive DNA-alkylating chemotherapeutics such as temozolomide and streptozotocin and those targeting DNA synthesis, such as gemcitabine and triapine, both of which act through inhibiting ribonucleotide reductase [12, 13]. Ribonucleotide reductase inhibitors decrease the synthesis of deoxyribonucleotides and thereby block DNA synthesis; they have often been investigated as a radiosensitisers [12]. Gemcitabine has recently been tested in combination with oxaliplatin for gastroenteropancreatic-/pan-NETs [14], while triapine is currently under investigation in ongoing phase 1 (NCT04234568) and phase 2 (NCT05724108) clinical trials for treating NET patients in combination with [^177^Lu]Lu-DOTA-TATE PRRT.

Here, we thus investigated whether treatment combinations with gemcitabine and triapine, particularly at metronomic levels, increased the therapeutic efficacy of [^177^Lu]Lu-DOTA-TATE in SSTR2-expressing and non-expressing cells. Metronomic chemotherapeutics here refers to the treatment in which low doses of anticancer drugs are given on a continuous or frequent, regular schedule. Alongside these drugs, another effective ribonucleotide reductase inhibitor, namely hydroxyurea [15–19], was also investigated for its potential to enhance the therapeutic efficacy of [^177^Lu]Lu-DOTA-TATE using the same strategy.

## Methods

### Cell culture

SSTR2A-expressing cells were generated as described previously [20]. Human osteosarcoma U2OS and U2OS + SSTR2A cells were maintained in Dulbecco’s Modified Eagle High-Glucose medium (DMEM-HG), whilst human pancreatic neuroendocrine BON1 and BON1 + SSTR2A cells were maintained in Dulbecco’s Modified Eagle Medium/Nutrient Mixture F-12 (DMEM/F-12) medium. Each medium was supplemented to 10% v/v foetal bovine serum (FBS), 2 mM L-glutamine and 1% v/v penicillin/streptomycin (100 units/mL and 100 µg/mL, respectively) to make DMEM-HG and DMEM/F-12 complete medium. BON1 + SSTR2A cells were additionally cultured with 0.5 mg/mL geneticin (Invivogen), added every other passage. Cell cultures were passaged with Accutase (Invitrogen) and grown in a humidified incubator at 37 °C and 5% CO_2_.

SSTR2A expression in cells was confirmed microscopically and by flow cytometry (Figure S1; see supplementary methods for details [7, 11]).

### Production of [^177^Lu]Lu-DOTA-TATE

For U2OS/U2OS + SSTR2A studies, post-clinical [^177^Lu]Lu-DOTA-TATE was supplied by Guy’s Radiopharmacy (Guy’s and St. Thomas’ NHS Foundation Trust) as Lutathera^®^ (Novartis). All studies were performed within 3 days of original patient treatment, with a volumetric activity calibrated to 370 MBq/mL on the day of patient administration; radiochemical yield and purity was > 98% and > 95%, respectively. For BON1/BON1 + SSTR2A studies, post-clinical [^177^Lu]Lu-DOTA-TATE was supplied the same day as patient treatments by Erasmus MC Radiopharmacy; this had been radiolabelled in-house using externally-sourced ^177^Lu (LuMark^®^, IDB Holland) to 53 MBq/nmol (radiochemical yield > 98%, radiochemical purity > 95%).

### [^177^Lu]Lu-DOTA-TATE uptake

1.5 × 10^5^ U2OS/U2OS + SSTR2A or 2 × 10^5^ BON1/BON1 + SSTR2A cells were seeded into 6-well plates in medium and allowed to adhere overnight in the incubator. The following day, 0.70–0.93 MBq (U2OS/U2OS + SSTR2A) or 0.75–1.03 MBq (BON1/BON1 + SSTR2A) [^177^Lu]Lu-DOTA-TATE was added to wells (25 nM total DOTA-TATE) and incubated for 24 h. Excess [^177^Lu]Lu-DOTA-TATE was then removed from wells and pooled together with two PBS washes (“Unbound” fraction). Cell-associated activity was harvested from wells by lysing cells with 0.5 M NaOH solution and pooled with a subsequent PBS wash (“Bound” fraction). Data was presented as percentage added activity (%AA) and activity per cell (Bq/cell).

### Viability studies after [^177^Lu]Lu-DOTA-TATE

2 × 10^3^ cells, both untreated and after 24 h [^177^Lu]Lu-DOTA-TATE treatment, were reseeded in medium-containing 96-well plates (180 µL/well final volume) and maintained in the incubator for 7 days. Medium was then aspirated, and cells were incubated for 4 h with 90 µL medium containing 3-(4,5-dimethylthiazol-2-yl)-2,5-diphenyl-2 H-tetrazolium bromide (MTT, 0.5 mg/mL) in the incubator. Excess MTT was then removed. Formazan crystals were dissolved with 50 µL/well in dimethylsulfoxide (DMSO) for 2 min at room temperature (RT) under gentle agitation. Well absorbance at 570 nm was then measured on a UV/V is absorbance microplate reader (SPECTROstar^®^ Nano, BMG LABTECH). Metabolic viability, following signal-to-background correction, was calculated as the percentage signal in treated cells normalised to the signal in untreated cells.

### Subcellular localisation of [^177^Lu]Lu-DOTA-TATE

Subcellular localisation of [^177^Lu]Lu-DOTA-TATE in cells followed the protocol for the uptake assays above, except that, following removal of excess [^177^Lu]Lu-DOTA-TATE, cells were incubated with ice-cold 0.1 M glycine (pH 2.5) for 10 min prior to pooling with a further PBS wash (“Membrane” fraction). Cells were then detached with Accutase and diluted in medium prior to pelleting and transferring the supernatant to the “Cytoplasm” fraction. The cell pellet was resuspended in ice-cold cell lysis buffer (25 mM KCl, 5 mM MgCl_2_, 10 mM Tris-HCl, and 0.5% v/v IGEPAL CA-630) and incubated for 15 min. Intact nuclei were pelleted and separated from lysed cells by centrifugation (10,000 *g*, 3 min) and the supernatant removed and combined with the previous portion of the “Cytoplasm” fraction. Pelleted nuclei were resuspended in phosphate-buffered saline (PBS) and transferred to tubes to gamma counting (“Nuclear” fraction). The percentage distribution of [^177^Lu]Lu-DOTA-TATE in cells was then calculated for the membrane, cytoplasm, and nucleus.

### Viability studies after chemotherapeutic agents

U2OS/U2OS + SSTR2A and BON1/BON1 + SSTR2A cells were seeded in 96-well plates at 2 × 10^3^ and 4 × 10^3^ cells/well, respectively, and allowed to adhere overnight in the incubator. Cells were then treated with a range of hydroxyurea, gemcitabine, and triapine concentrations for 24 h. Hydroxyurea in U2OS/U2OS + SSTR2A cells was used at 152 µg/mL for 24 h [21]. Chemotherapeutics were then removed, and cells washed with PBS prior to returning to the incubator in fresh medium. Cells were then grown for a further 5 days post-chemotherapeutics prior to assessing metabolic viability, as described above. Chemotherapeutic concentrations needed to inhibit population growth by 50% (GI_50_) were then calculated.

### [^177^Lu]Lu-DOTA-TATE uptake and effect on viability post chemotherapeutics

1.5 × 10^5^ U2OS/U2OS + SSTR2A or 2 × 10^5^ BON1/BON1 + SSTR2A cells were seeded into 6-well plates in DMEM-HG or DMEM/F-12 medium, respectively, and allowed to adhere overnight in the incubator. Cells where then treated with chemotherapeutics for 24 h at the following concentrations: 152 µg/mL hydroxyurea, 4 ng/mL gemcitabine, and 200 ng/mL triapine for U2OS/U2OS + SSTR2A cells; and 152 µg/mL hydroxyurea, 2 ng/mL gemcitabine, and 100 ng/mL triapine for BON1/BON1 + SSTR2A cells. Following a 4 h recovery period after chemotherapeutics, medium was replaced with 1 mL of medium containing [^177^Lu]Lu-DOTA-TATE for 24 h at 0.7–0.93 MBq for U2OS/U2OS + SSTR2A cells or 0.75–1.03 MBq for BON1/BON1 + SSTR2A cells. Untreated control wells had the equivalent volume of PBS diluent. Uptake and viability studies were then carried out as above.

### Cell cycle, SSTR2A, and size changes post chemotherapeutics

All studies were performed in cells at 4 h after removal of each chemotherapeutics treatment.

For cell cycle studies, cells were pulse-labelled with medium containing 20 µM 5-ethynyl-2’-deoxyuridine (EdU, Click-iT™ Plus EdU Flow Cytometry Assay Kit, Invitrogen) for 1 h prior to harvesting by Accutase detachment. Cells were then fixed, permeabilized, and blocked before 30-minute incubation at RT following manufacturer instructions and labelled with Alexa Fluor™-488/-647-labelled-picolyl azide. Cells were further washed, resuspended in 4′,6-diamidino-2-phenylindole (DAPI; 1:1,000 dilution in PBS) and stained for 20 min prior to dilution in blocking solution and analysis (BD LSRFortessa™). Results were analyzed using FlowJo (v10.9.0).

For SSTR2A expression studies, 1.5 × 10^5^ U2OS/U2OS + SSTR2A and BON1/BON1 + SSTR2A cells were seeded in 6-well plates and placed in the incubator to adhere overnight. Cells were treated with chemotherapeutics for 24 h at the same concentrations stated above. Determination of SSTR2A expression levels by flow cytometry post chemotherapeutics was performed as described in the Supplementary methods.

For cell size studies, cells were detached by Accutase, washed with PBS and resuspended in medium prior to live-cell analysis by flow cytometry (BD LSRFortessa™). General cell size information was acquired using the forward-scatter laser.

### Statistics

Data are presented as mean ± standard deviation. Statistical analysis was carried out using GraphPad Prism v9.1.0 and differences were deemed statistically significant at p-values < 0.05. In uptake and viability studies, the Kruskal-Wallis analysis of variance (ANOVA) test was carried out to determine statistical significance. One-way ANOVAs comparing all chemotherapeutics against untreated in a cell line were used in viability studies.

## Results

### Baseline uptake of radioactivity and viability in cells treated with [^177^Lu]Lu-DOTA-TATE, [^177^Lu]LuCl_3_, or DOTA-TATE

It was determined that [^177^Lu]Lu-DOTA-TATE uptake per cell was maximal at 25 nM with a 24 h incubation period (Figure S2); these conditions were therefore used for all studies below. Baseline uptake assays under these conditions confirmed the SSTR2-targeting nature of [^177^Lu]Lu-DOTA-TATE (Table 1). The varying SSTR2A expression between U2OS + SSTR2A and BON1 + SSTR2A cells levels affected [^177^Lu]Lu-DOTA-TATE uptake (*p* = 0.0158), with higher uptake observed in U2OS + SSTR2A (17.8 ± 2.4%, 216.9 ± 47.4 mBq/cell) than in BON1 + SSTR2A cells (9.6 ± 0.6%, 111.2 ± 16.5 mBq/cell). Both parental U2OS and BON1 cells showed negligible uptake. Uptake of unchelated [^177^Lu]LuCl_3_ in U2OS/U2OS + SSTR2A cells was also low in cells with no notable differences induced by SSTR2A expression (Figure S3).

**Table 1:** Uptake of [^177^Lu]Lu-DOTA-TATE after 24-hour incubation (25 nM DOTA-TATE, 0.74–1.03 MBq) as percentage added activity (%AA) and mBq/cell in U2OS and BON1 parental and U2OS + SSTR2A- and BON1 + SSTR2A-expressing cell lines. Also shown is the percentage metabolic viability in cells at day 7 post incubation with [^177^Lu]Lu-DOTA-TATE. *N* = 3–4

Cell line	Uptake (%AA)	Uptake (mBq/cell)	Viability (%)
U2OS	0.3 ± 0.0	4.4 ± 1.0	102.2 ± 11.9
U2OS + SSTR2A	17.8 ± 2.4	216.9 ± 47.4	59.5 ± 22.3
BON1	0.5 ± 0.3	6.5 ± 6.3	91.2 ± 4.5
BON1 + SSTR2A	9.6 ± 0.6	111.2 ± 16.5	45.9 ± 10.4

Both U2OS + SSTR2A and BON1 + SSTR2A cells showed significantly reduced metabolic viabilities compared to their parental counterparts following [^177^Lu]Lu-DOTA-TATE incubation (Table 1). Metabolic viabilities were calculated as 59.5 ± 22.3% for U2OS + SSTR2A cells and 45.9 ± 10.4% for BON1 + SSTR2A cells. In comparison, parental U2OS and BON1 cells incubated with [^177^Lu]Lu-DOTA-TATE or SSTR2A-expressing cells incubated with [^177^Lu]LuCl_3_ showed only minor reductions in metabolic viability (Table 1, Figure S4). Similarly, treatment of U2OS + SSTR2A cells with non-radioactive DOTA-TATE for 24 h did not reduce metabolic viability (Figure S5).

### Chemotherapeutic GI_50_ concentrations

**Fig. 1:**
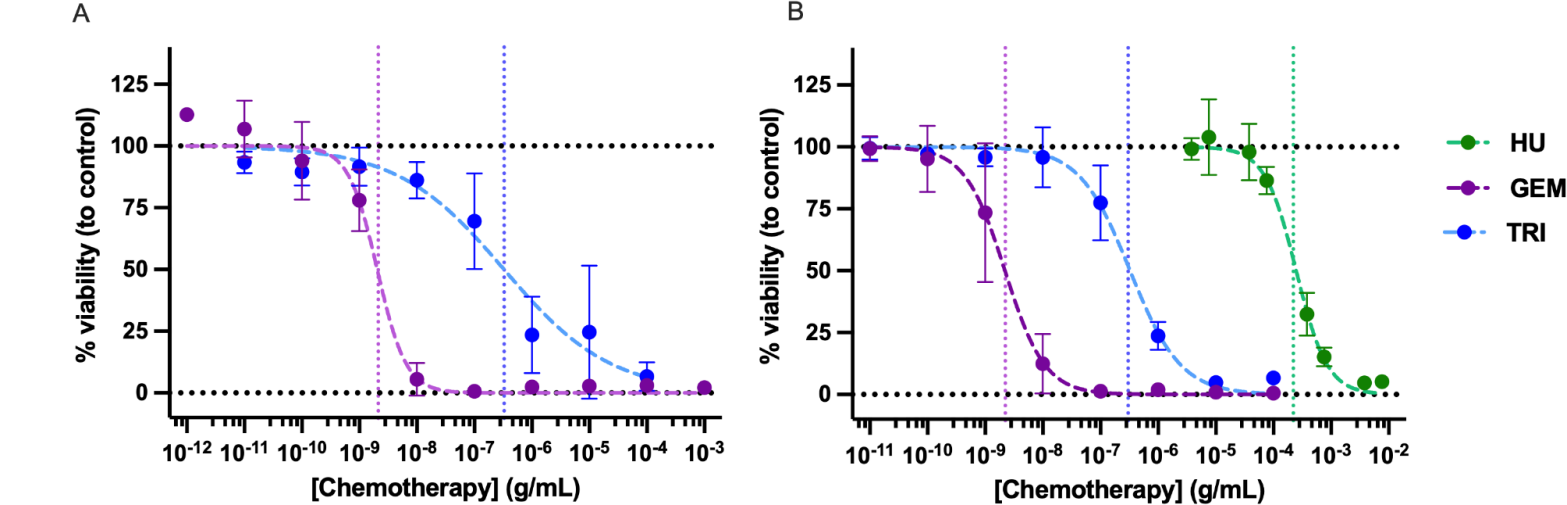
Chemotherapeutic viability curves of U2OS + SSTR2A (**A**) and BON1 + SSTR2A (**B**) cells. Dotted vertical lines = Growth Inhibition GI_50_ values for each chemotherapeutic, calculated from the respective fitted curves. Treatments were for 24-hour with hydroxyurea (HU), gemcitabine (GEM) and triapine (TRI); determined by the MTT assay. *N* = 3. GI_50_ values for HU in U2OS + SSTR2A cells were taken from the literature and validated [21]

The hydroxyurea, gemcitabine and triapine concentrations at which cell metabolic viability was reduced to 50% compared to control (GI_50_) were determined for U2OS + SSTR2A and BON1 + SSTR2A cells (Fig. 1; Table 2, Figure S6). GI_50_ metronomic values were calculated to be 4.7 ng/mL and 120.5 ng/mL in U2OS + SSTR2A cells for gemcitabine and triapine, respectively, and 248.1 µg/mL, 2.2 ng/mL and 326.7 ng/mL in BON1 + SSTR2A cells for hydroxyurea, gemcitabine and triapine, respectively. As such, the following concentrations were used: 152 µg/mL (2 mM) hydroxyurea as per [21], 4 ng/mL gemcitabine and 200 ng/mL triapine for U2OS/U2OS + SSTR2A cells; and 152 µg/mL hydroxyurea, 2 ng/mL gemcitabine and 100 ng/mL triapine for BON1/BON1 + SSTR2A cells.

**Table 2:** GI_50_ values for hydroxyurea, gemcitabine and triapine in U2OS and BON1 parental and U2OS + SSTR2A- and BON1 + SSTR2A-expressing cell lines. Values used for further studies are in brackets. GI_50_ values for hydroxyurea in U2OS + SSTR2A cells were taken from the literature

Cell line	Hydroxyurea (µg/mL)	Gemcitabine (ng/mL)	Triapine (ng/mL)
U2OS + SSTR2A	152 (152)	4.7 (4)	120.5 (200)
BON1 + SSTR2A	248.1 (152)	2.2 (2)	326.7 (100)

### [^177^Lu]Lu-DOTA-TATE uptake, subcellular localisation, and cell viability post chemotherapeutics

**Fig. 2:**
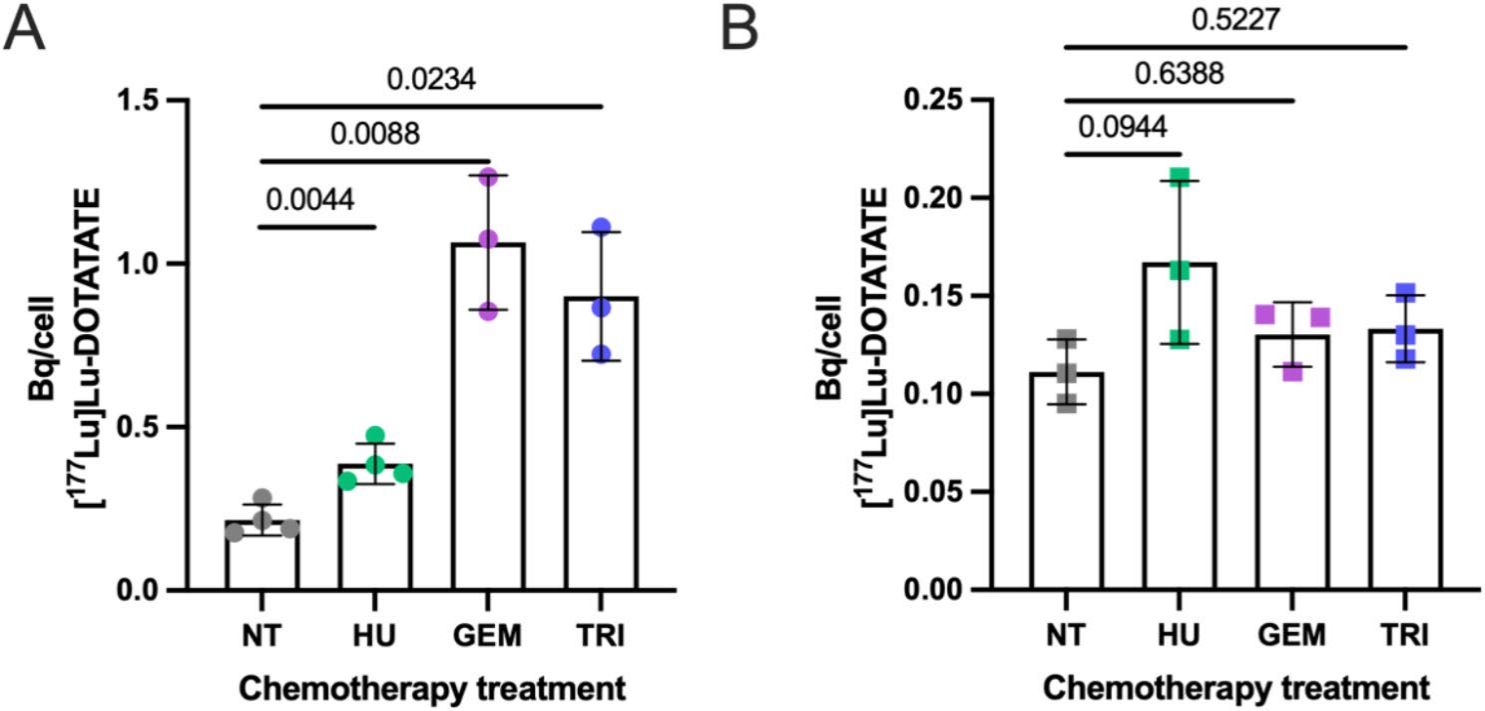
Uptake in Bq/cell of [^177^Lu]Lu-DOTA-TATE (25 nM DOTA-TATE, 0.7–1.03 MBq) after 24-hour incubation in U2OS + SSTR2A (**A**) and BON1 + SSTR2A (**B**) cells post treatment with hydroxyurea (HU), gemcitabine (GEM) and triapine (TRI). NT = not chemotherapeutic treated. Numbers above graphs refer to P-values

Pre-treatment of U2OS + SSTR2A cells with hydroxyurea, gemcitabine, and triapine enhanced [^177^Lu]Lu-DOTA-TATE uptake (Fig. 2). Internalized quantities of [^177^Lu]Lu-DOTA-TATE significantly increased from 0.2 ± 0.05 Bq/cell in untreated U2OS + SSTR2A cells to 0.4 ± 0.1, 1.1 ± 0.2, and 0.9 ± 0.2 Bq/cell when pre-treated with hydroxyurea, gemcitabine, or triapine, respectively. In BON1 + SSTR2A cells, differences were also seen but were non-significant (*p* = 0.13). Subcellular localization remained mostly cytoplasmic in all cells, both with and without chemotherapeutics (Tables 3 and 4). For example, 93.5 ± 0.9% and 94.9 ± 0.2% of internalized [^177^Lu]Lu-DOTA-TATE was cytoplasmic in untreated and hydroxyurea-pre-treated U2OS + SSTR2A cells, respectively. The percentage of activity in the cytoplasm was lower in U2OS + SSTR2A cells treated with gemcitabine and triapine, namely 86.2 ± 6.9% and 83.8 ± 7.8%, respectively. Cytoplasmic localization of [^177^Lu]Lu-DOTA-TATE in BON1 + SSTR2A cells remained consistent whether pre-treated with or without chemotherapeutics (Table 4).

**Table 3:** Subcellular localisation of [^177^Lu]Lu-DOTA-TATE in U2OS + SSTR2A cells post chemotherapeutic treatment as percentage of total uptake. *N* = 3

Cell line	Membrane (%)	Cytoplasm (%)	Nucleus (%)
Untreated	4.1 ± 0.6	93.5 ± 0.9	2.3 ± 0.4
Hydroxyurea	3.0 ± 0.5	94.9 ± 0.2	2.2 ± 0.4
Gemcitabine	10.0 ± 6.6	86.2 ± 6.9	3.8 ± 0.4
Triapine	11.8 ± 8.0	83.8 ± 7.8	4.4 ± 0.2

**Table 4:** Subcellular localisation of [^177^Lu]Lu-DOTA-TATE in BON1 + SSTR2A cells post chemotherapeutic treatment as percentage of total uptake. *N* = 3

Cell line	Membrane (%)	Cytoplasm (%)	Nucleus (%)
Untreated	7.3 ± 1.0	90.2 ± 1.7	2.5 ± 0.7
Hydroxyurea	6.3± 1.6	91.4± 1.3	2.3 ± 0.4
Gemcitabine	6.3 ± 3.2	90.5 ± 4.6	3.2 ± 1.4
Triapine	5.2 ± 1.9	92.1 ± 2.7	2.7 ± 0.9

**Fig. 3:**
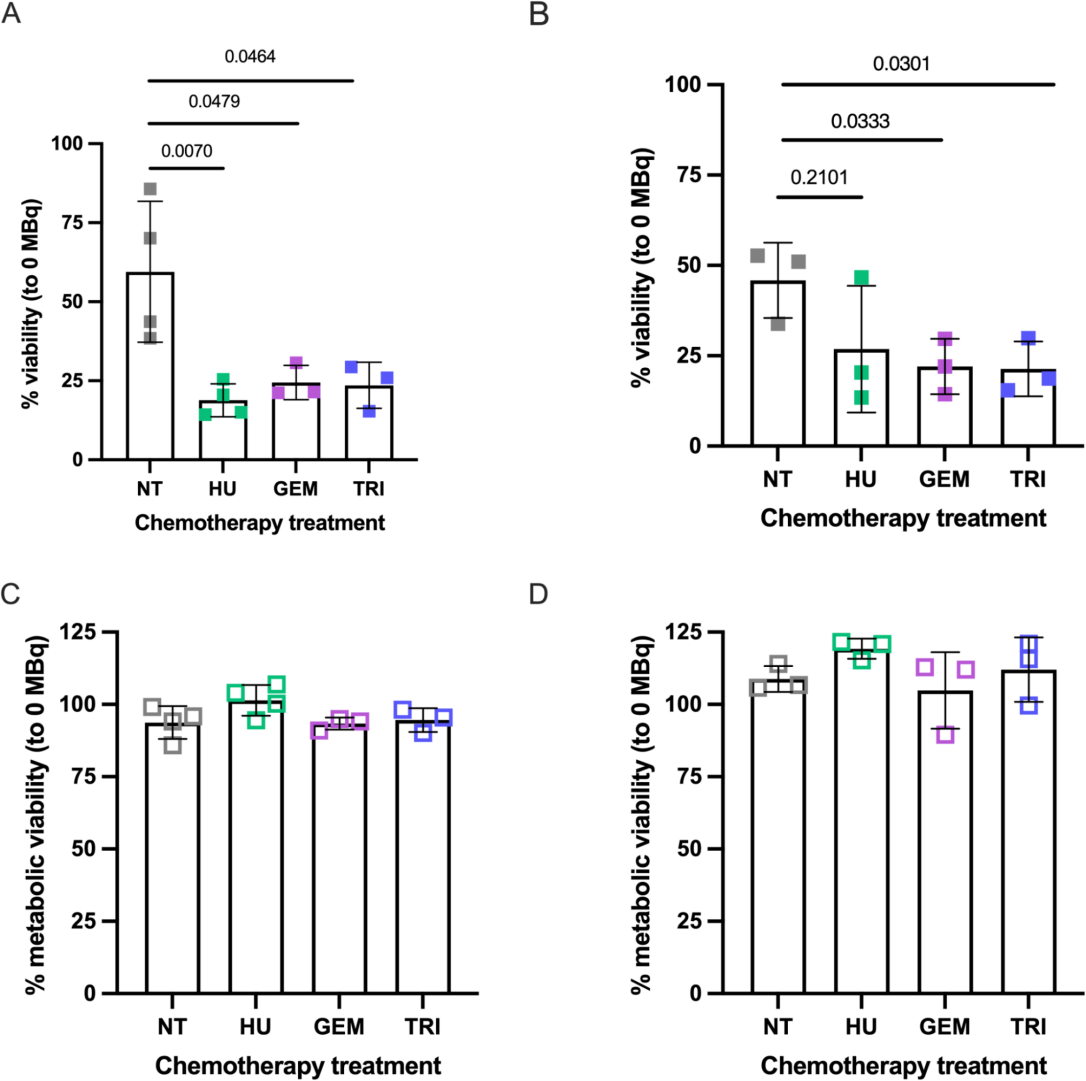
Percentage metabolic viability in U2OS + SSTR2A (**A**) and BON1 + SSTR2A (**B**) cells, pre-treated with hydroxyurea (HU), gemcitabine (GEM) and triapine (TRI), at day 7 post incubation with [^177^Lu]Lu-DOTA-TATE. [^177^Lu]Lu-DOTA-TATE incubation was for 24 h. Viability studies were also carried out in U2OS (**C**) and BON1 (**D**) parental cells. [^177^Lu]Lu-DOTA-TATE concentrations were set at 25 nM total DOTA-TATE (0.7–1.03 MBq). NT = not chemotherapeutically treated. *N* = 3

Cell viability following [^177^Lu]Lu-DOTA-TATE incubation also decreased further with hydroxyurea, gemcitabine, and triapine pre-treatment in both U2OS + SSTR2A and BON1 + SSTR2A cells (Fig. 3). For example, the percentage viability of U2OS + SSTR2A cells decreased from 59.5 ± 22.3% when incubated with [^177^Lu]Lu-DOTA-TATE alone to 18.8 ± 5.2%, 24.5 ± 5.4%, and 23.6 ± 7.3% in hydroxyurea, gemcitabine or triapine pre-treated cells, respectively.

Combining hydroxyurea, gemcitabine or triapine with [^177^Lu]Lu-DOTA-TATE in parental U2OS and BON1 cells did not affect viability, highlighting the requirement of internalization of [^177^Lu]Lu-DOTA-TATE to see potentiation of it through other therapies (Fig. 3). However, potentiation of radiation effects was seen for U2OS and U2OS + SSTR2A cells pre-treated with hydroxyurea, gemcitabine or triapine before X-ray radiation (Figure S7), confirming the radiosensitizing effects of the chemotherapeutics in these cells.

### Effect of chemotherapeutics on cell cycle, SSTR2A expression and cell size

**Fig. 4:**
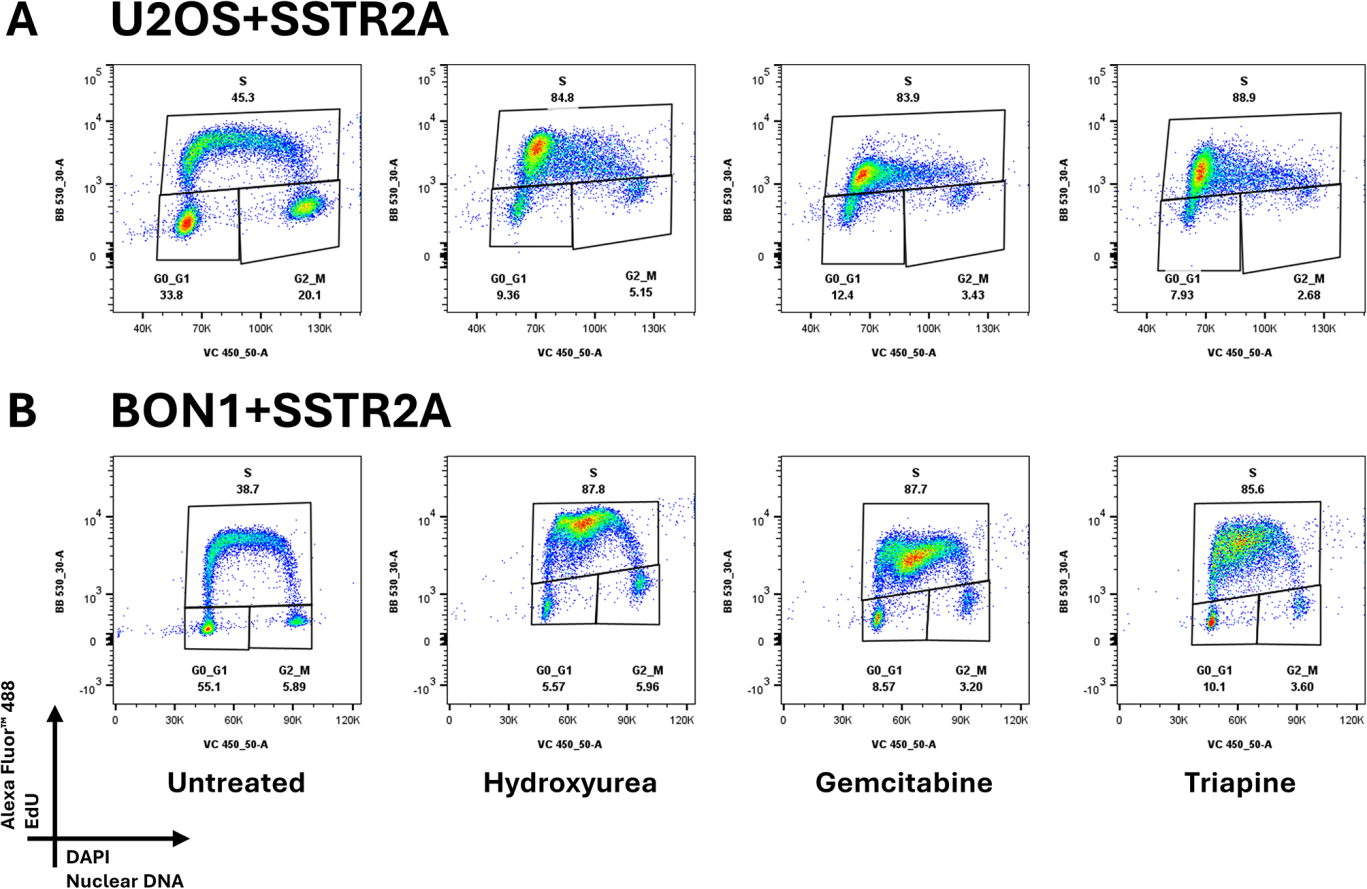
Representative flow cytometry density plots of the cell cycle in U2OS + SSTR2A- and BON1 + SSTR2A-expressing cells when left untreated or treated with hydroxyurea, gemcitabine or triapine. EdU: 5-Ethynyl-2′-deoxyuridine

**Fig. 5:**
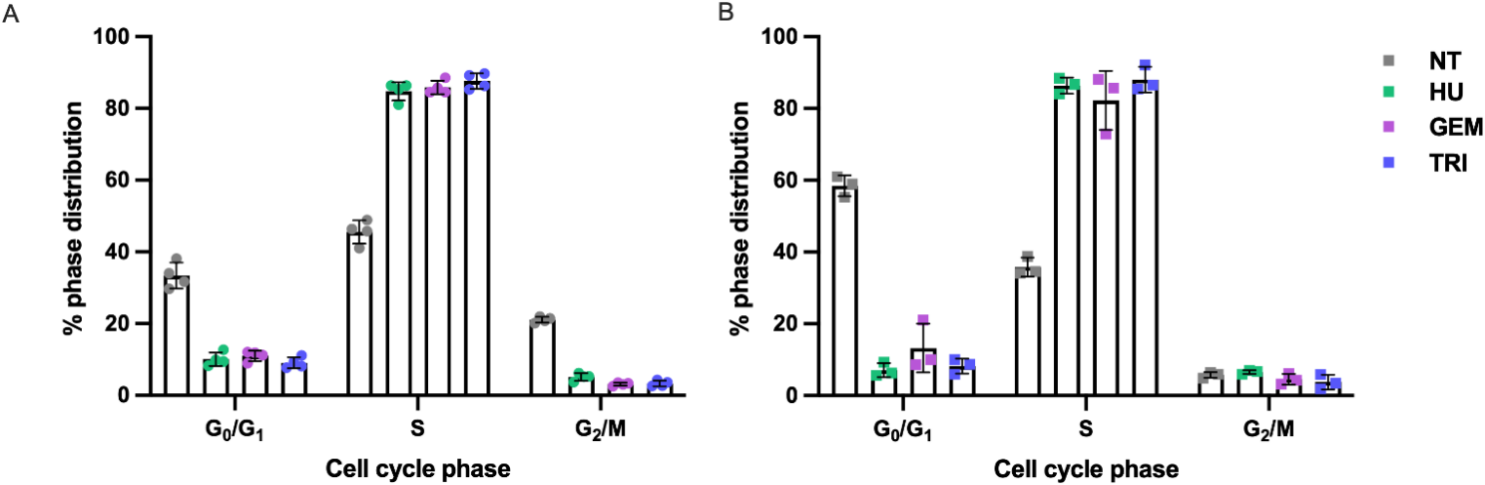
Percentage of U2OS + SSTR2A (**A**) and BON1 + SSTR2A (**B**) cells in the G_0_/G_1_, S and G_2_/M phases of the cell cycle following a 24-hour chemotherapeutic treatment with hydroxyurea (HU), gemcitabine (GEM) and triapine (TRI); determined by flow cytometry. NT = not chemotherapeutic treated. *N* = 3

Hydroxyurea, gemcitabine and triapine induced a larger proportion of cells in the S-phase for both U2OS + SSTR2A and BON1 + SSTR2A cell cultures than in untreated cultures (Figs. 4 and 5). More specifically, the S-phase sub-population of asynchronous U2OS + SSTR2A cell cultures was increased from 45.5 ± 3.3% to 84.8 ± 2.5%, 85.9 ± 1.9%, and 86.6 ± 2.2% after treatment with hydroxyurea, gemcitabine, and triapine, respectively. For asynchronous BON1 + SSTR2A cell cultures, the S-phase sub-population increased from 35.8 ± 2.6% to 86.4 ± 2.2%, 82.2 ± 8.2% and 88.0 ± 3.6% after treatment with hydroxyurea, gemcitabine, and triapine, respectively.

**Fig. 6:**
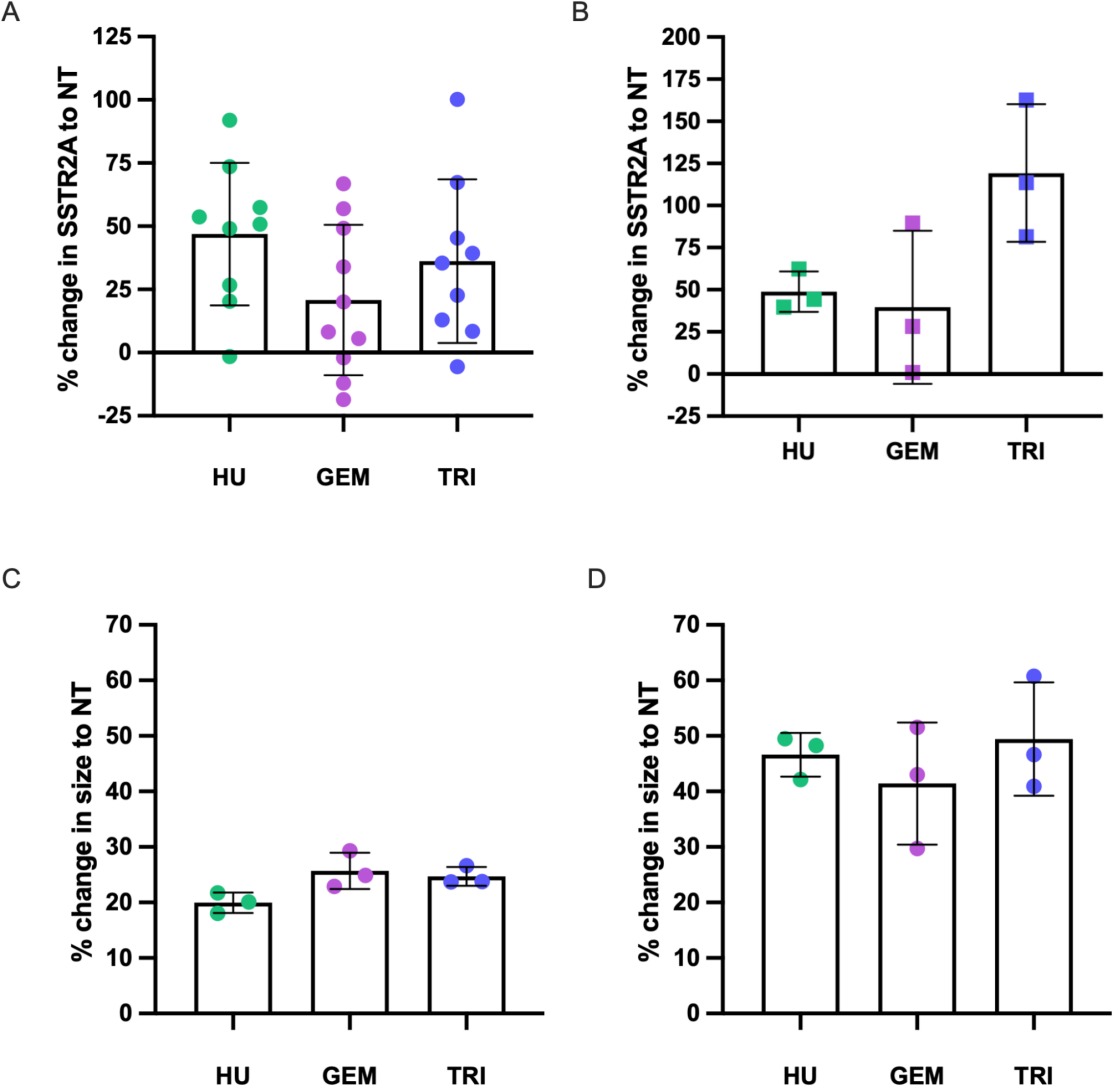
Changes in SSTR2A expression of U2OS + SSTR2A (**A**) and BON1 + SSTR2A (**B**) cells following a 24-hour chemotherapeutic treatment with hydroxyurea (HU), gemcitabine (GEM) and triapine (TRI) relative to non-treated (NT) cells; determined by flow cytometry (*N* = 3–9). Changes in cell size of U2OS + SSTR2A (**C**) and BON1 + SSTR2A (**D**) cells following chemotherapeutics; also determined by flow cytometry. All studies were conducted 4 h after chemotherapeutic treatment was completed. *N* = 3

Each chemotherapeutic was also found to increase SSTR2A expression (Fig. 6A-B). For U2OS + SSTR2A cells, mean increases in SSTR2A expression were 46.9 ± 28.2%, 20.8 ± 29.7%, and 36.2 ± 32.4%, while for BON1 + SSTR2A cells mean increases were calculated at 48.8 ± 12.0%, 39.6 ± 45.4% and 119.3 ± 40.9% from baseline expression levels for cells when pre-treated with hydroxyurea, gemcitabine, and triapine, respectively. Chemotherapeutic treatments also increased cell size (Fig. 6C-D). For example, forward-scatter laser median fluorescence intensity for U2OS + SSTR2A cells treated with hydroxyurea, or triapine were 121,835 ± 1,738, 127,616 ± 799 or 126,635 ± 962, respectively, whereas baseline median fluorescence intensity was 101,568 ± 2,081. Cell size did depend on the cell cycle phase (Figure S8), with cells in G1 showing the smallest size and cells in late S-phase (after DNA duplication) showing the largest cell size in BON1 + SSTR2A cells. It is also noted that relative cell sizes of chemotherapeutic pre-treated cells, at all phases of the cell cycle, were consistently larger than cells from baseline cultures.

## Discussion

Here, studies were performed with U2OS/U2OS + SSTR2A and BON1/BON1 + SSTR2A cells, which have previously been useful to study the therapeutic efficacy of [^177^Lu]Lu-DOTA-TATE. Here, using transfected as well as parental cell lines also provided an opportunity to investigate targeted direct effects from ^177^Lu as well as off-target exposure of cells to free [^177^Lu]LuCl_3_. Our work determined that pre-treating neuroendocrine cancer cells with metronomic levels of the radiosensitizing DNA-synthesis inhibitors hydroxyurea, gemcitabine, or triapine enhanced the effectiveness of [^177^Lu]Lu-DOTA-TATE PRRT in both U2OS + SSTR2A and BON1 + SSTR2A cells. Changes in [^177^Lu]Lu-DOTA-TATE uptake and therapeutic efficacy were not observed in control conditions, here referring to unchelated [^177^Lu]LuCl_3_ and non-radioactive, unlabelled DOTA-TATE. This highlights the specificity of [^177^Lu]Lu-DOTA-TATE to the SSTR2A receptor as well as the dependence on radioactive ^177^Lu and its requirement for this to be cell-bound and internalized so as to exert a cytotoxic effect in NET cells.

Pre-treatment of U2OS + SSTR2A cells with hydroxyurea, gemcitabine, or triapine enhanced cellular uptake of [^177^Lu]Lu-DOTA-TATE, increasing by double or more than observed at baseline levels. Correspondingly, metabolic viability was decreased in cells when [^177^Lu]Lu-DOTA-TATE was combined with each chemotherapeutic. Our work complements previous studies carried out by Nayak et al. who similarly reported an enhanced uptake of SSTR2A-targeting [^177^Lu]Lu-DOTA-TOC in cells pre-treated with gemcitabine, which was linked to a reduced cell viability compared to cells treated with [^177^Lu]Lu-DOTA-TOC alone [22]. The mechanism behind the potentiation of [^177^Lu]Lu-DOTA-TATE by these chemotherapeutics remains unclear though and our work does not determine whether this enhanced treatment efficacy is synergistic or additive. Now that initial concentrations of chemotherapeutics have been established, future combination index studies can be performed to determine whether the effects of chemotherapeutics and [^177^Lu]Lu-DOTA-TATE are additive or synergistic in nature.

Here, we also found that cytoplasmic localization of [^177^Lu]Lu-DOTA-TATE remained unaltered. This suggests that there is no significant change in trafficking of [^177^Lu]Lu-DOTA-TATE upon cellular interaction following chemotherapeutics; therefore it can be determined that subcellular localization was not a significant determining factor in the potentiation observed for [^177^Lu]Lu-DOTA-TATE cytotoxicity when combined with the chemotherapeutics used.

The ribonucleotide reductase inhibitors, hydroxyurea, gemcitabine, and triapine, have also previously been described as radiosensitisers [12]. This effect was observed here in both U2OS and U2OS + SSTR2A cells; indeed, all three chemotherapeutics sensitised cells to X-ray radiation. Whilst it is possible that the chemotherapeutics radiosensitised cells to [^177^Lu]Lu-DOTA-TATE PRRT by enhancing the likelihood of unrepaired DNA damage as it did for X-rays, here, we showed that they also increased levels of SSTR2A expression. Work using other chemotherapeutics, namely 5-fluorouracil, temozolomide and streptozotocin, also reported increased SSTR2A expression in NET cells and enhanced [^177^Lu]Lu-DOTA-TATE uptake and cytotoxicity compared to [^177^Lu]Lu-DOTA-TATE monotherapy [23]. It is therefore likely that alongside other factors described below, enhanced SSTR2A expression is a main driver for increased [^177^Lu]Lu-DOTA-TATE uptake and therefore toxicity. Whether the same would hold true in cancer cells that express SSTR2A naturally, remains to be seen. As SSTR2A expression is naturally seen at lower levels in a variety of healthy tissues also [24, 25], it would also be of great interest to determine whether similar enhancements in receptor expression post chemotherapeutics are tumour-specific or happen in healthy tissues too. This could enhance the probability of normal tissue complications and thereby influence maximal administered [^177^Lu]Lu-DOTA-TATE activities for this therapeutic strategy. By using transfected models, even non-NET cells, this work contributes knowledge towards the feasibility of the metronomic chemotherapeutic/PRRT combination approach in scenarios where SSTR2 expression is high or low. Further work in this area would enable us to determine whether, and importantly, at what point, there may be a cut-off for therapeutic benefit for such a combination approach.

As well as affecting SSTR2A expression, a larger proportion of cells remained in S-phase after having been treated with the chemotherapeutics. This is not uncommon as various therapies have been shown to delay progression through the S-phase. The availability of sister chromatids and consequently homologous recombination repair, particularly during late S-phase contributes to a relative radioresistance [26, 27], and therefore it is unlikely that the enhanced cytotoxicity observed in the combination therapy studies here is solely attributable to the cell cycle phase. Another observation in the studies carried out here is an increase in the size of cells treated with the chemotherapeutics. Cells naturally increase in size as they progress through the cell cycle from G_1_ to S and G_2_/M. In addition to this natural variation in cell size, chemical inhibition of cellular progression into or through the S-phase with agents such as those employed here are known to introduce a mismatch in metabolic balance, through continued RNA and protein synthesis during this temporary inhibition [15, 28], creating the same trend in cell sizes, but at an elevated level at all phases of the cell cycle compared to those observed at baseline. The significance and impact of this on radiosensitisation remains unclear. However, increases in overall cell size, and therefore changes in subcellular compartment size (and [^177^Lu]Lu-DOTA-TATE localisation within them), could influence the accessibility of SSTR2A receptors and consequently the amount of [^177^Lu]Lu-DOTA-TATE that binds (separately to enhanced SSTR2A expression). This in turn could affect the amount internalised into cells and/or the biological effectiveness of [^177^Lu]Lu-DOTA-TATE. As mentioned, the exact influence of cells size remains unclear and to determine the influence of cell size of cytotoxicity from [^177^Lu]Lu-DOTA-TATE requires further work and cellular dosimetry calculations.

There are very few studies looking into metronomic chemotherapeutic and PRRT combination therapies, although the Lu-X trial shows that adding a chemotherapeutic at metronomic levels with PRRT is a valid approach that minimizes toxicity [29]. But it remains difficult to compare our work to clinical trials such as Lu-X where capecitabine was combined with [^177^Lu]Lu-DOTA-TATE in patient with neuroendocrine tumours [29], as chemotherapy is administered at regular intervals in parallel and surrounding the first/final [^177^Lu]Lu-DOTA-TATE administrations. However, in our study, we investigated the acute effects of this combination strategy. Although promising, the in vitro data acquired here needs to be validated in preclinical studies and the optimal regimen needs to be determined. The chemotherapeutic concentrations used here were chosen based on their ability to modulate SSTR2A expression and cell size close to the respective chemotherapeutics’ GI_50_ values. Further studies should test more concentrations per chemotherapeutic, particularly those lower than used here, and with a further variety of [^177^Lu]Lu-DOTA-TATE incubation activities. Also, it would be interesting to determine whether other chemotherapeutics have a similar effect on [^177^Lu]Lu-DOTA-TATE uptake and cytotoxicity in cells, particularly with the proposed combination strategy, and to ascertain through which mechanism that is realised, e.g. cell cycle phase/synchronisation, modulation of SSTR2A receptor expression, or a mixture thereof. Initial clinical studies would also be needed to determine whether (and for how long) existing chemotherapeutic schedules enhance SSTR2A expression and consequently [^177^Lu]Lu-DOTA-TATE uptake; this could be achieved using either ex vivo tumour samples or through PET imaging using [^68^Ga]Ga-DOTA-TATE, for example. Next, clinical combination studies to maximize [^177^Lu]Lu-DOTA-TATE tumour uptake will need to be carried out, albeit it with a view to ensure that toxicities from this approach remain acceptable. It could be envisaged that administered activities of [^177^Lu]Lu-DOTA-TATE, when used as part of combination regimen, can be lowered without compromising overall tumour uptake when compared to its administration as a monotherapy. Considering the chemotherapeutics investigated here are already in use in the clinic, with triapine also currently in active phase I and II clinical trials, it is forecast that any such approach combining [^177^Lu]Lu-DOTA-TATE with hydroxyurea, gemcitabine, or triapine has a relatively low barrier to adoption in the clinic and yet could greatly increase outcome for patients with NETs.

## Conclusions

Hydroxyurea, gemcitabine and triapine all increased SSTR2A expression and [^177^Lu]Lu-DOTA-TATE uptake whilst selectively reducing cell metabolic viability in transfected U2OS + SSTR2A and BON1 + SSTR2A cells compared to [^177^Lu]Lu-DOTA-TATE monotherapy. Further investigations could transform patient care and positively increase outcomes for patients treated with [^177^Lu]Lu-DOTA-TATE.

### Electronic supplementary material

Below is the link to the electronic supplementary material.

Supplementary Material 1

## Data Availability

Data is available upon reasonable request to the corresponding author.

## Abbreviations

[^177^Lu]Lu-DOTA-TATE: [^177^Lu]Lu-[DOTA^0^-Tyr^3^]octreotate
ANOVA: Analysis of variance
DAPI: 4′,6-diamidino-2-phenylindole
DMEM/F-12: Dulbecco’s Modified Eagle Medium/Nutrient Mixture F-12
DMEM-HG: Dulbecco’s Modified Eagle High-Glucose medium
DMSO: Dimethylsulfoxide
EDU: 5-ethynyl-2’-deoxyuridine
FBS: Foetal bovine serum
GEM: Gemcitabine
HU: Hydroxyurea
MTT: 3-(4,5-dimethylthiazol-2-yl)-2,5-diphenyl-2 H-tetrazolium bromide
NET: Neuroendocrine tumour
PBS: Phosphate-buffered saline
PPRT: Peptide receptor radionuclide therapy
RT: Room temperature
SSTR2A: Somatostatin receptor 2 A
TRI: Triapine
