# Supplementary material for: Enhancing [177Lu]Lu-DOTA-TATE therapeutic efficacy in vitro by combining it with metronomic chemotherapeutics
Source: EJNMMI Res. 2024 Aug 13;14:73. doi: 10.1186/s13550-024-01135-0 (PMC11322472; doi:10.1186/s13550-024-01135-0)
Supplement: Supplementary file 1 — Supplementary Material 1 [file 13550_2024_1135_MOESM1_ESM.docx]

**SUPPLEMENTARY INFORMATION**

**SUPPLEMENTARY METHODS**

***SSTR2A expression***

Both widefield fluorescence microscopy and flow cytometry were used to determine SSTR2A expression in U2OS/U2OS+SSTR2A cells and BON1/BON1+SSTR2A cells.

For widefield fluorescence microscopy, 1x10^4^ U2OS/U2OS+SSTR2A cells and 3x10^4^ BON1/BON1+SSTR2A cells were seeded in wells of a black-walled, clear-bottom 24-well plate (Greiner Bio-One) containing 1 mL warm DMEM-HG or DMEM/F-12 media, respectively. Cells were allowed to attach for 2 days at 37°C and 5% CO_2_. Following adherence and growth, cell membranes were stained with a fluorescent dye through replacing the original culture media with warmed, fresh media containing a 1:200 dilution of CellBrite® Orange Cytoplasmic Membrane Dye (Biotium Inc., cat. #30022). Cells were returned to the incubator for 30 minutes to stain membranes, followed by two washes with PBS prior to fixation with 4% v/v paraformaldehyde (PFA) for 10 minutes at room temperature (RT). Following fixation, PFA was removed, and wells washed twice with PBS before blocking cells with 2% w/v BSA in PBS for 1 hour at RT. Following blocking, 2% w/v BSA was aspirated, and cells were incubated overnight at 4°C with 1:400 dilution (in 2% w/v BSA) of recombinant anti-somatostatin receptor 2 antibody (UMB1: C-terminal, rabbit monoclonal, Abcam, ab134152). The following day, excess antibody was removed, and wells washed twice with 2% w/v BSA solution, followed by an incubation with 1:1000 dilution (in 2% w/v BSA) of goat anti-rabbit IgG secondary antibody (Alexa Fluor™-488, Abcam) for 1 hour at RT. After aspirating wells of excess secondary antibody, wells were washed twice with 2% w/v BSA solution prior to incubation for 3 minutes at RT with 1:1000 dilution (in PBS) of DAPI (Sigma-Aldrich). After aspiration, wells were washed and filled with PBS and stored at 4°C in the dark. Images were acquired with an EVOS™ M5000 imaging system (Thermo Scientific™) equipped with a 3.2 MP monochrome CMOS camera and EVOS™ fluorite/phase-contrast objectives (4x to 40x magnification). Fluorescent signal was detected using the installed DAPI (Ex: 357/44 nm; Em: 447/60 nm), GFP (Ex: 470/22 nm; Em: 525/50 nm) and RFP (Ex: 531/40 nm; Em: 593/40 nm) LED cubes. Images were acquired at a magnification of 20x, with a pixel resolution of 0.311 µm/pixel. Post-acquisition image viewing and processing (background removal, colour re-allocation and montage creation) was performed using ImageJ (Fiji distribution, version 1.54).

For flow cytometry analysis of SSTR2A expression, cells (1x10^6^) were detached from culture vessels and pelleted by centrifugation (1,000 g for 2 min) and the media aspirated. Cells were then resuspended in 4% v/v PFA and fixed at RT for 10 min. After, cells were pelleted by centrifugation and excess PFA aspirated prior to resuspension in 0.5% v/v Triton™ X-100 (Thermo Scientific™) and permeabilised at RT for 10 minutes. Cells were once again pelleted by centrifugation and excess Triton™ X-100 removed prior to resuspension in blocking solution (PBS containing 2.5 mM EDTA and 2% w/v BSA). Cells were stained with UMB1 primary antibody (1:400 dilution, Abcam) for 30 minutes at RT before pelleting cells and aspirating excess antibody. Cells were washed once with blocking solution prior to repeating pelleting and aspiration. Cells were then resuspended and incubated with Alexa Fluor™ 647 secondary antibody (1:1000 dilution, Abcam) for 30 minutes at RT, protected from light. After incubation, excess antibody was removed prior to a final wash with blocking solution. Cells analysed using the BD LSRFortessa™. Results are expressed as the median fluorescence intensity (MFI) of the overall acquired signal, analysed using FlowJo version 10.9.0 (BD, USA).

***[^177^Lu]Lu-DOTA-TATE receptor saturation uptake***

1.5x10^5^ U2OS+SSTR2A cells were seeded in technical duplicate wells of 6-well plates and placed in the incubator, allowing cells to adhere and grow overnight. An additional triplicate set of wells were seeded for estimating population size at the end of the assay by manual haemocytometer counting. The following day, the media was replaced with 1 mL of media containing the [^177^Lu]Lu-DOTA-TATE dilutions for final total DOTA-TATE concentrations ranging from 0.1 to 75 nM. Plates were then returned to the incubator for 24 h prior to further manipulation. The following day, excess [^177^Lu]Lu-DOTA-TATE was removed from wells and pooled together with two PBS washes (“Unbound” fraction). Cell-associated activity was harvested from wells by lysing cells with 0.5 M NaOH solution and pooled with a subsequent PBS wash (“Bound” fraction). Data is presented as both percentage uptake of total added activity and normalised as Bq/cell uptake.

***[^177^Lu]LuCl_3_ uptake***

1.5x10^5^ U2OS and U2OS+SSTR2A cells were seeded into 6-well plates in DMEM-HG DMEM-HG and allowed to adhere overnight in the incubator. The following day, 0.97 MBq [^177^Lu]LuCl_3_ was added to wells and incubated for 24 hours. Excess [^177^Lu]LuCl_3_ was then removed from wells and pooled together with two PBS washes (“Unbound” fraction). Cell-associated activity was harvested from wells by lysing cells with 0.5 M NaOH solution and pooled with a subsequent PBS wash (“Bound” fraction). Data is presented as both percentage uptake of total added activity and normalised as Bq/cell uptake.

***[^177^Lu]LuCl_3_ and DOTA-TATE viability***

2x10^3^ U2OS or U2OS+SSTR2A cells, after treatment for 24 hours with [^177^Lu]LuCl_3_ (0.97 MBq) or DOTA-TATE (25 nM, U2OS+SSTR2A only), were reseeded in technical sextuplet wells in 96-well plates containing a final media volume of 180 µL/well. Dedicated wells with no cells present served as background signal controls. Plates were placed in the incubator and cells allowed to adhere and grow for 7 days. After 7 days, DMEM-HG was aspirated and replaced with 90 µL of basal DMEM-HG containing 0.5 mg/mL MTT. Plates were returned to the incubator for 4 h prior to aspiration of excess MTT. Formazan crystals were then dissolved with 50 µL/well DMSO for 2 minutes at RT under gentle agitation. Well absorbance at 570 nm wavelength was then immediately measured on a UV/Vis absorbance microplate reader (SPECTROstar® Nano, BMG LABTECH). Metabolic viability, following signal background-correction, was calculated as the percentage of signal normalized to the signal of the respective fully-untreated or chemotherapeutic-alone cells.

***X-ray radiotherapy viability post chemotherapeutics***

1.5x10^5^ U2OS or U2OS+SSTR2A cells were seeded into 6-well plates in DMEM-HG and allowed to adhere overnight in the incubator. Cells where then treated with chemotherapeutics for 24 hours at 2 mM HU, 4 ng/mL GEM and 200 ng/mL TRI. Following chemotherapeutics, media was refreshed, and cells were X-ray irradiated at 0 (sham-irradiated), 2 and 4 Gy using the image-guided small animal radiotherapy system, SmART+, X-ray irradiator (Precision X-ray Irradiation). The calibrated dose-rate was 6.1796 Gy.min^-1^; the X-ray beam used for treatment was set at a voltage of 225 kV, a current of 20 mA and passed through a 0.3 mm Cu filter. Viability analyses were then carried out as above.

**SUPPLEMENTARY FIGURES**


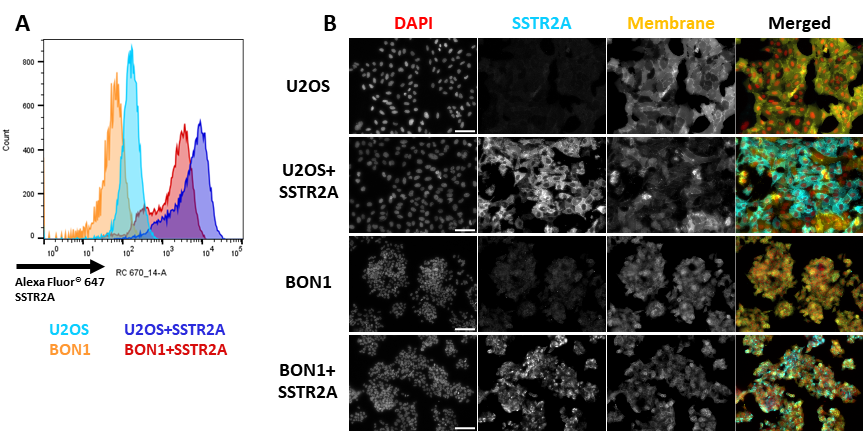


Figure S1. SSTR2A immunostaining of U2OS/U2OS+SSTR2A and BON1/BON1+SSTR2A cells for flow cytometry analysis (A) and immunofluorescent microscopy (B). Microscopy images were acquired at x20 magnification (pixel resolution = 0.311 pixels/µm). White scale-bar in DAPI column (bottom right) = 100 µm. Membrane staining was carried out using the CellBrite® orange cytoplasmic membrane dye.

**
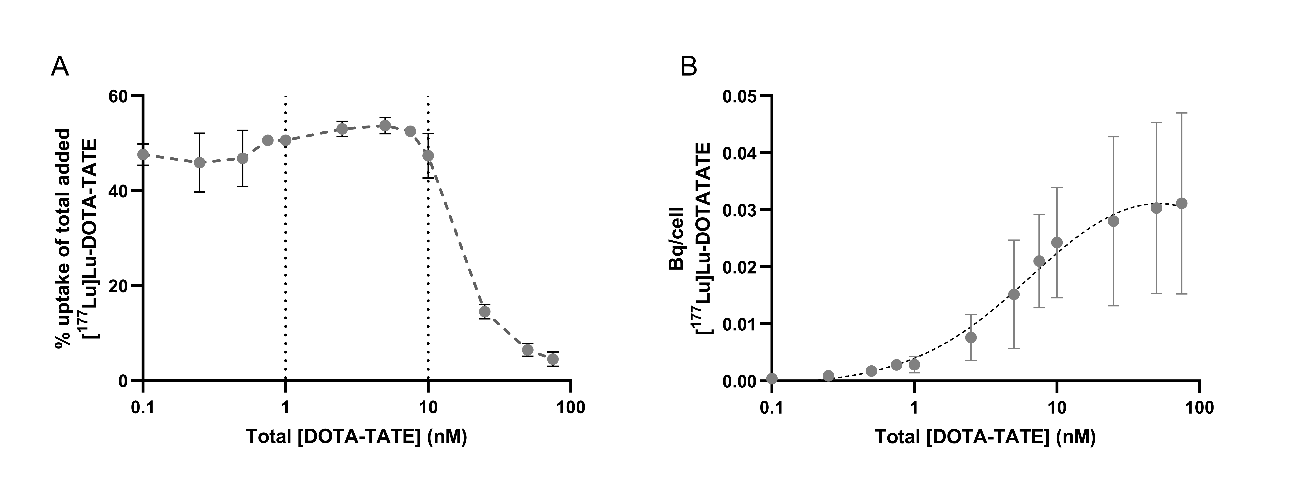
**

**Figure S2.** Percentage (**A**) and Bq/cell uptake (**B**) of [^177^Lu]Lu-DOTA-TATE set at a single 24 h exposure across 12 DOTA-TATE concentrations between 0.1 to 75 nM. N=3.


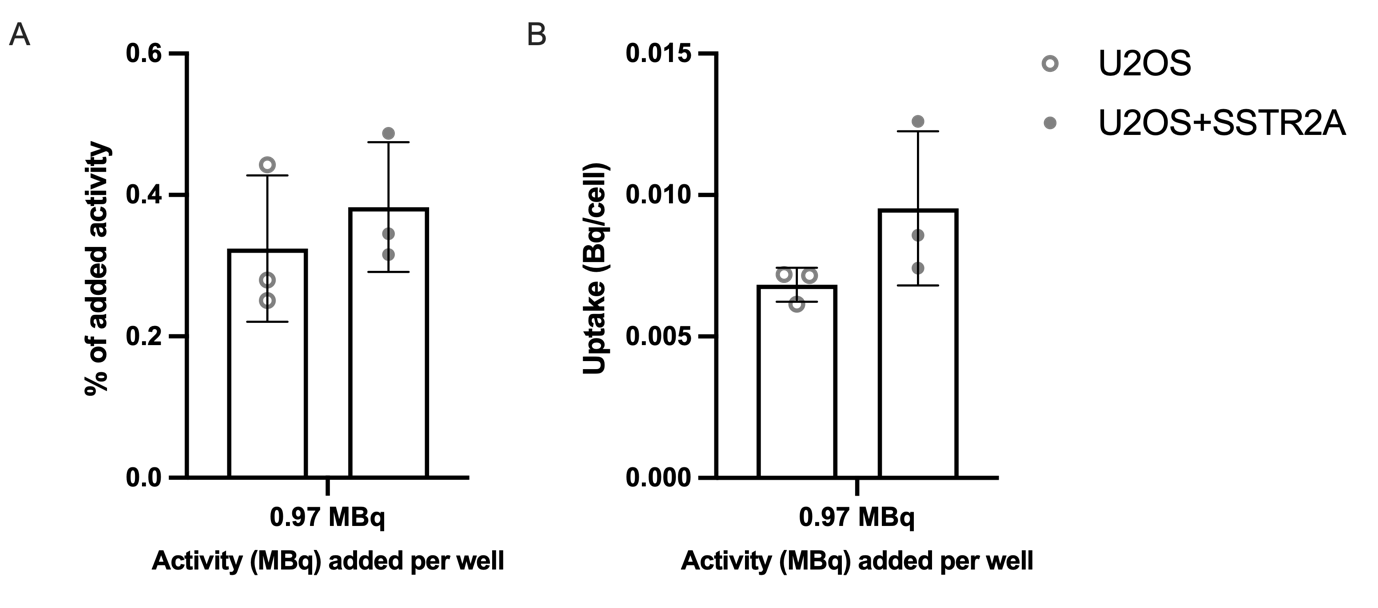


**Figure S3.** Uptake of [^177^Lu]LuCl_3_ (0.97 MBq) after 24-hour incubation in U2OS parental (**A**) and SSTR2A-expressing (**B**) cell lines, displayed as percentage of total added activity and Bq/cell. N=3.

**
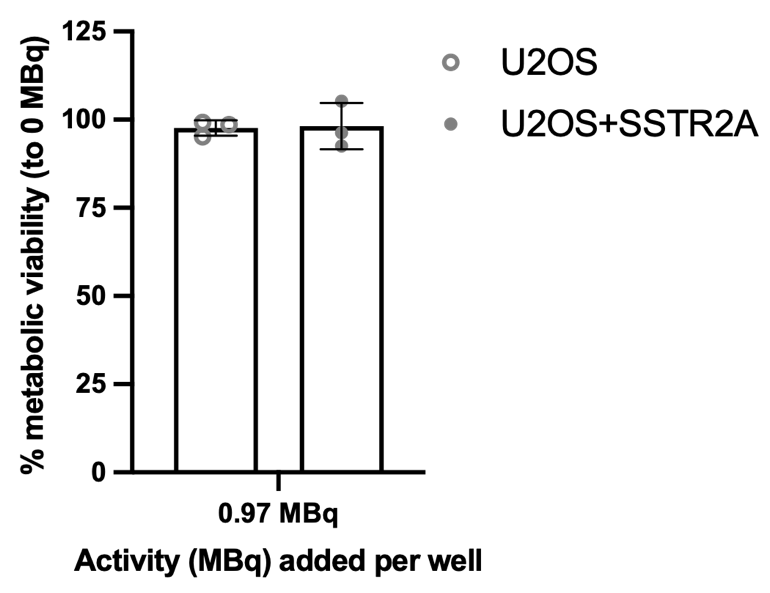
**

**Figure S4.** Metabolic viability of U2OS parental and SSTR2A-expressing cell lines after treatment with [^177^Lu]LuCl_3_ (0.97 MBq) for 24 hours. N=3.

**Figure S5.** Metabolic viability of U2OS+SSTR2A cells after treatment with unlabelled DOTA-TATE (25 nM) for 24-hours. N=4.


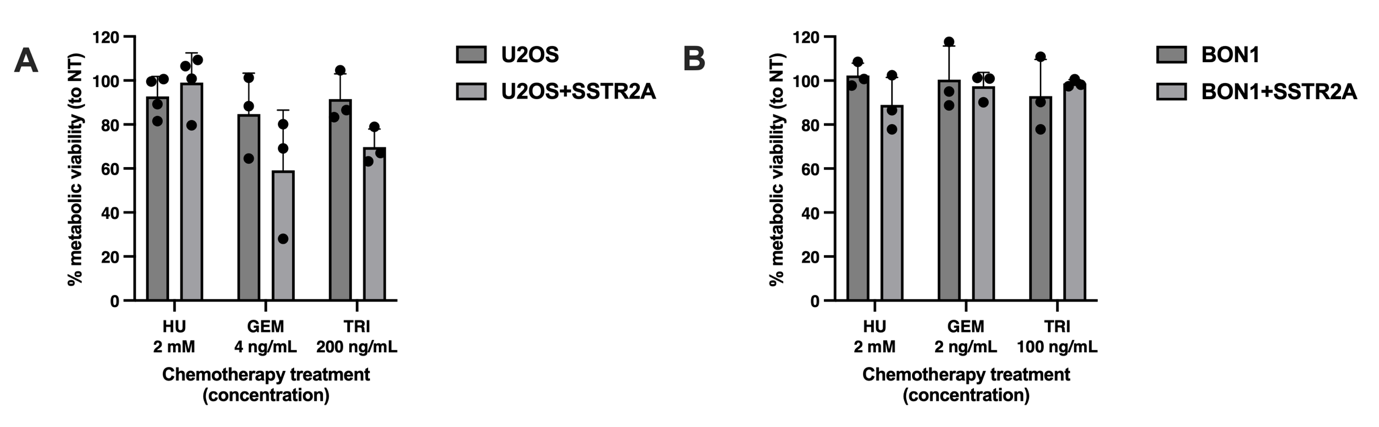


**Figure S6.** Percentage metabolic viabilities of U2OS and BON1 parental and SSTR2A-expressing cell lines treated with hydroxyurea (HU), gemcitabine (GEM) and triapine (TRI) alone at day 7 post treatment. Values were normalised to non-treated (NT) values, which were set to 100%. N=3.

**
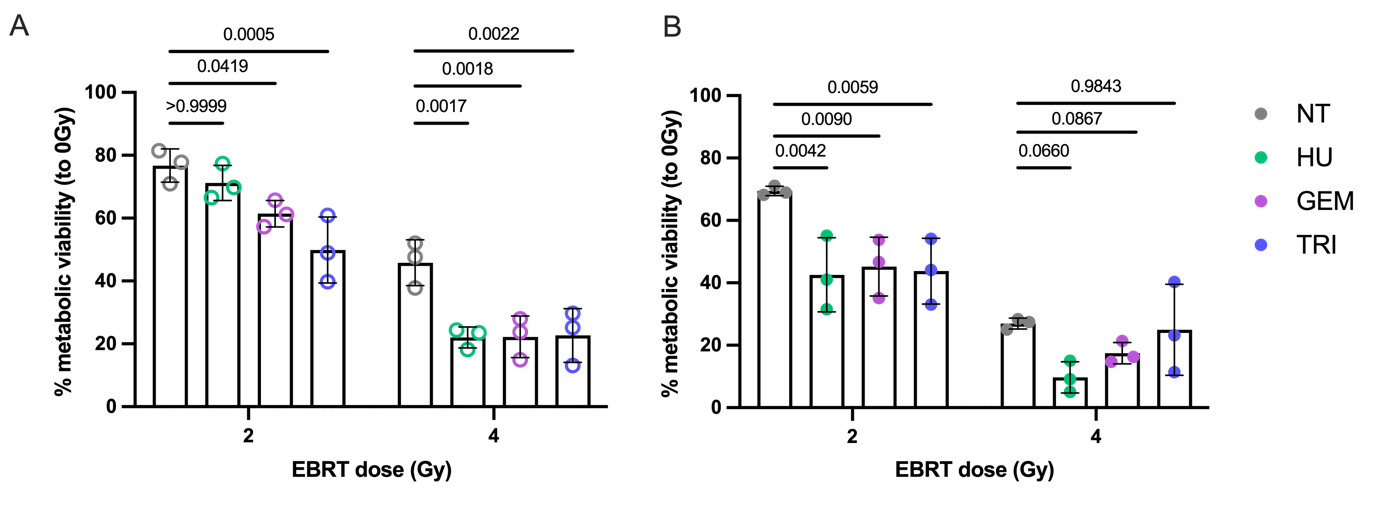
**

**Figure S7.** Percentage metabolic viabilities of U2OS parental (**A**) and SSTR2A-expressing (**B**) cell lines pretreated with hydroxyurea (HU), gemcitabine (GEM) and triapine (TRI) followed by treatment with X-ray radiation 4 hours later at 0, 2 and 4 Gy. NT = not chemotherapeutic treated. EBRT = external beam radiotherapy, i.e. X-rays. Values were normalised to untreated, 0 Gy values. N=3. Data was analysed by 2-way ANOVA.


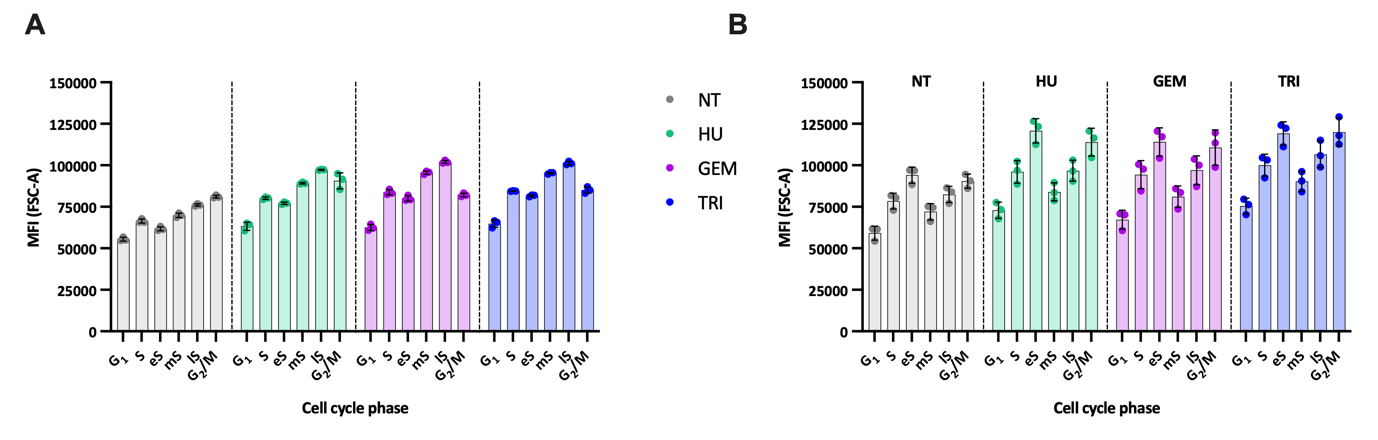


**Figure S8.** Mean fluorescence intensity (MFI) for forward scatter (FSC-A) values, as a measure of cell size, against cell cycle phase (G1, S, and G_2_/M) as determined by flow cytometry for U2OS+SSTR2A (A) and BON1+SSTR2A (B) cells. eS is early S-phase; mS is mid S-phase; lS is late S-phase. N=3.
